# Imported Retail Beef and Chicken Meat Products Serve as Reservoirs for Emerging Antibiotic‐Resistant Pathotypes of *Escherichia coli* in Pristine Areas Free From Agricultural Activity

**DOI:** 10.1002/mbo3.70273

**Published:** 2026-03-30

**Authors:** Saehah Yi, Kathleen A. Alexander, Auja Bywater, Galaletsang Dintwe, Ashton N. Sies, Thomas H. Haidl, Andrew D. S. Cameron, Monica A. Ponder

**Affiliations:** ^1^ Department of Food Science and Technology Virginia Tech Blacksburg Virginia USA; ^2^ CARACAL/Chobe Research Institute Kasane Botswana; ^3^ Department of Fisheries and Wildlife Virginia Tech Blacksburg Virginia USA; ^4^ Department of Biology University of Regina Regina SK Canada; ^5^ Institute for Microbial Systems and Society, Faculty of Science University of Regina Regina SK Canada

**Keywords:** Botswana, EPEC, ExPEC, foodborne pathogens, plasmids, sub‐Saharan Africa

## Abstract

Increasing attention is being directed toward the role of retail meat in introducing pathogens and antibiotic‐resistant bacteria into local food supplies. This study characterized the antibiotic resistance (AR) and virulence of *E. coli* isolates from chicken and beef (*n* = 109) imported for retail sale in Kasane, Botswana. In this relatively pristine environment, commercial beef and chicken production is absent, resulting in reliance on imports, creating concerns that multidrug‐resistant (MDR) and pathogenic *E. coli* may be introduced through the food supply sourced from distant regions. *E. coli* was isolated from 54.7% of samples (63/109). Antibiotic susceptibility testing against a panel of 12 antibiotics revealed resistance to 11 antibiotics, with multiple combinations of resistance phenotypes identified. Higher levels of MDR were found in chicken isolates (45.5%) compared to beef (13.3%), with the highest resistance rates observed for tetracycline, trimethoprim/sulfamethoxazole, and doxycycline. Genomic analysis of eight MDR isolates revealed diverse sequence types, including diarrheagenic and extraintestinal‐associated serotypes. The latter has critical implications in health systems where this clinical presentation may not be investigated with foodborne pathogen exposures. Plasmid‐borne AR genes with conjugation‐associated genes were detected in most isolates, suggesting that some AR genes may be horizontally transferable by plasmid conjugation. Several isolates clustered with human and chicken isolates from around the globe, highlighting the high potential for retail beef and chicken products to harbor MDR pathogenic *E. coli*, including emerging pathogens, and to introduce those microbes and associated AR attributes into new ecosystems.

## Graphical abstract

Retail meat–derived *E. coli* frequently harbored multidrug‑resistance phenotypes, diverse virulence determinants, and plasmid‑encoded resistance genes with high conjugative potential. Results indicate meat and poultry as underrecognized reservoirs facilitating dissemination of antimicrobial resistance and pathogenic lineages, with imported products enabling long‑distance transmission of agricultural AR risks even in geographically remote regions.

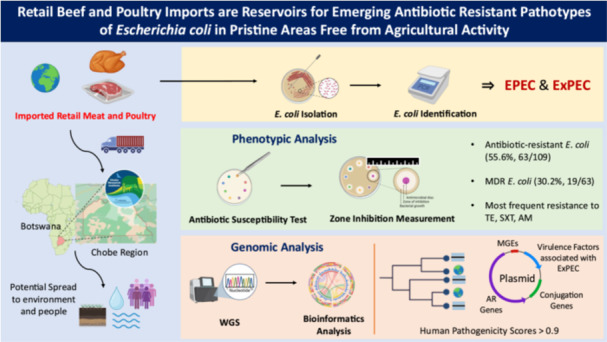

## 1 Introduction

The global rise of antimicrobial resistance (AMR) is one of the most important public health challenges of the 21st century (WHO 2023). AMR threatens the effectiveness of antimicrobial agents essential for treating infectious diseases in humans, animals, and agriculture (Prestinaci et al. 2015). Recent estimates suggest that by 2050, AMR could be associated with up to 8 million deaths annually worldwide, with the heaviest burden projected in Africa, followed by Asia (WHO 2021a; Murray et al. 2022). Antibiotic resistance (AR) represents a major and clinically important component of the broader AMR burden (Murray et al. 2022; CDC 2025). The rise in AR corresponds with higher mortality rates in vulnerable populations who are more susceptible due to a higher prevalence of immunosuppressive conditions such as HIV (Liu et al. 2025). This is particularly concerning for low‐ and middle‐income countries, where surveillance efforts are limited and access to essential antibiotics may be limited. Antibiotic‐resistant bacteria (ARB), including pathogens, have been isolated from food products (Ballash et al. 2021). These bacteria do not necessarily cause disease upon consumption but may increase the risk of colonization or infection under conditions of compromised food hygiene, or serve as a reservoir for resistance genes that can be transferred to other bacteria (Jans et al. 2018; Grudlewska‐Buda et al. 2023). Among the food commodities, meat is considered one of the highest‐risk sources of ARB, with evidence of elevated levels of phenotypic resistance in meat‐derived bacterial isolates (Jans et al. 2018; Farrukh et al. 2025).

In some regions, including parts of sub‐Saharan and Southern Africa, antibiotics have historically been used in high concentrations for metaphylaxis and treatment of infectious diseases in beef and chicken production, applying selective pressure on the gut microbiota of livestock and driving the development and persistence of ARB within the gastrointestinal tract (Van Boeckel et al. 2019; Andrew Selaledi et al. 2020; Kimera et al. 2020; Odey et al. 2023; Islam et al. 2024). Comprehensive data on antibiotic use in animals are limited in many African countries, as surveillance and reporting efforts are often inconsistent. However, recent studies suggest widespread use of antibiotics in animal production systems, and consequently, a high prevalence of ARB in animal products (Van Boeckel et al. 2019). Across Sub‐Saharan Africa, more than 50% of isolates obtained from poultry have been reported to be resistant to commonly used antibiotics, including tetracyclines, sulfonamides, penicillins, and other agents associated with multidrug resistance (Van Boeckel et al. 2019; Azabo et al. 2022). These findings highlight the continued presence of ARB in animal‐source food products, despite global efforts to reduce antibiotic use in animal husbandry, and underscore the importance of ongoing resistance monitoring, especially in regions where surveillance data remain limited.

*Escherichia coli* is of particular interest in AR research due to its widespread presence in the gastrointestinal tract of humans and animals (Partridge et al. 2018). *E. coli* is widely used as an indicator organism for fecal contamination in food and water, and its presence in food products, particularly meat, is a critical food safety concern (Deblais et al. 2025). During slaughter and meat processing, *E. coli* from the animal's intestines can contaminate carcass surfaces through contact with fecal matter, especially in settings with poor hygiene practices (Dharma et al. 2022). Pathogenic *E. coli* strains, including O157:H7, have also been isolated from meat products, with many showing resistance to multiple antibiotic classes (Magwira et al. 2005). Although monitoring for AR *E. coli* from meats in sub‐Saharan Africa is infrequent, a few studies have characterized antibiotic resistance genes and mobile genetic elements (MGEs) in foodborne bacteria in this region. Ingle et al. (2018) reported extensive AR gene carriage among enteropathogenic *E. coli* (EPEC) isolated from children in sub‐Saharan Africa, with many AR genes co‐located on plasmids, integrons, and transposons (Ingle et al. 2018). Similarly, Nnah et al. (2025) identified diverse MGEs, including integrons, insertion sequences, and IncF‐type plasmids, as key contributors to the spread of AR in *E. coli* isolated from food sources across Africa.

Globally, *E. coli* harboring virulence‐associated and antibiotic resistance–associated factors in meat and poultry‐derived products are recognized as emerging threats to public health and may facilitate the introduction of disease into both human and wildlife population (Wasiński 2019). *E. coli* strains show considerable genetic diversity and can be classified into distinct pathotypes based on their virulence characteristics and clinical associations. Broadly, these include intestinal pathogenic *E. coli* (IPEC), which are major causes of foodborne diarrheal illness and outbreaks globally (Pakbin et al. 2021). Beef is a frequent reservoir for IPEC, most notably Shiga toxin *E. coli* O157:H7 (Alhadlaq et al. 2024). Poultry, particularly chickens, can be a source of extra‐intestinal pathogenic *E. coli* (ExPEC), including avian pathogenic *E. coli* (APEC) associated with coccobacillosis disease in poultry, as well as uropathogenic *E. coli* (UPEC) linked to urinary tract infections, sepsis, and neonatal meningitis in humans (Shaik et al. 2017; Wasiński 2019). Pathogenic *E. coli* often harbor virulence factors related to adhesion, immune evasion, and iron acquisition (Barrios‐Villa et al. 2018; Monroy‐Pérez et al. 2024), and are commonly related to well‐characterized serotypes (e.g., O25:H4 and O18:H7) and multidrug‐resistant lineages (Shaik et al. 2017; Barrios‐Villa et al. 2018).

This study aims to characterize *E. coli* isolates from beef and chicken products sold in the Chobe region of Botswana by analyzing their antibiotic resistance phenotypes, virulence genes, and MGEs. The Chobe region, located in northern Botswana, is ecologically distinct due to its extensive protected area and dry lands, which limit commercial livestock and chicken production for meat and create a relatively pristine environment with minimal direct agricultural influence and low local selection pressure for antibiotic resistance in food animals. As a result, most retail beef and chicken meat consumed in the Chobe region is sourced from other parts of Botswana or South Africa. This unique context raises concerns that AR or pathogenic strains may be introduced into the local food system with imported meat products. This study hypothesizes that *E. coli* isolates from meat in this setting may carry resistance and virulence traits similar to those identified in regional and international studies, suggesting potential for both local dissemination and broader transmission via imported food products. By applying whole‐genome sequencing (WGS), this study seeks to identify emerging pathotypes and resistance determinants in *E. coli* and assess the potential role of MGEs in shaping ABR characteristics within the food supply.

## 2 Methods

### 2.1 Sample Collection and *E. coli* Isolation

Beef and chicken products were obtained monthly from January to December 2022 from two different grocery stores in Kasane, Botswana. In total, 109 retail meat samples were collected, including 47 beef samples and 62 chicken samples. Based on information provided by retail vendors, the products were sourced either from South Africa or from other regions of Botswana outside the Chobe region. All collected samples were processed within 24 h of collection. Fifty grams of chicken feet or chicken pieces were weighed into sterile filter bags, and 50 mL of 0.1% peptone buffer was added. For the egg sample, the shell was excluded, and the yolk and albumen were mixed with 50 mL of 0.1% peptone buffer. Similarly, for beef products, 50 grams of mince, ox tripe, beef bones, and/or bone meal were weighed into a sterile filter bag and mixed with 100 mL of 0.1% peptone. For chicken products, fifty grams of chicken intestines (mala) were processed by passing 30mLs of sterile peptone through the lumen using a sterile syringe and the resulting liquid was collected into a filter bag containing the intestine pieces. Once processed, all diluted samples were mixed using an orbital shaker (Oxford Lab Products, San Diego, California) for 10 min at 2000 rpm.

After mixing, the peptone rinse from the processed samples was decanted into two 15 mL Falcon tubes and centrifuged (Axiology Labs, Gauteng, South Africa) at 3000 rpm for 10 min. The supernatant was discarded, and the pellet was resuspended in 13mLs of the appropriate selective media: Brilliant Green Bile Broth (BGBB) (Becton, Dickinson and Company) for *E. coli* (ThermoFisher Scientific, Waltham, Massachusetts). BGBB enrichments were plated onto Eosin Methylene Blue (EMB) (Becton, Dickinson and Company, Sparks, Maryland) and incubated at 37°C for 24 h. Colonies with a green sheen, indicative of *E. coli*, were streaked for isolation on EMB, followed by 4 single colony passages on MacConkey Agar (Becton, Dickinson and Company), where bright pink colonies were selected. Only purified multiply passaged isolates were shipped to Virginia Tech to comply with U.S. Department of Agriculture Animal and Plant Health Inspection Services, Veterinary Services requirements (Permit 610‐22‐272‐03465) to prevent importation of viruses that would threaten US livestock.

### 2.2 PCR Detection of *E. coli* Genes

Presumptive *E. coli* were initially enriched and isolated using MacConkey and EMB agars, which selectively support the growth of lactose‐fermenting enteric bacteria and therefore increase the likelihood of recovering *E. coli*. Detection of the *phoA* gene was subsequently performed using established primer sets targeting alkaline phosphatase (Kong et al. 1999). Although *phoA* is not strictly specific to E. coli, potential cross‐detection of other Enterobacteriaceae was minimized through prior selective enrichment, differential plating, and repeated single‐colony purification before PCR confirmation. A multiplex PCR was used to amplify key virulence genes: *eae* designed to detect Enterohemorrhagic and Enteropathogenic pathotypes, and *est*1b to detect Enterotoxigenic *E. coli* (Vidal et al. 2005). The sizes of the expected mPCR products and the concentrations of the primer pairs are listed in Supporting Information S1: Table 1. The mPCR amplification was conducted in a total reaction volume of 25 μL containing 12.5 μL of Gotaq Green Master Mix 2X, 1 μL of Magnesium chloride 25 μM, 7.5 μL of Nuclease‐Free Water, 2 μL of DNA template, and the corresponding primers. Nuclease‐free water was added in place of the DNA template to obtain a negative control. DNA from Enterohemorrhagic *E. coli* O157:H7 ATCC 43895 (ATCC) and Enterotoxigenic *E. coli* H10407 (Serotype O78:H11; NR‐4, BEI Resources, NIAID, NIH, USA) were used as positive controls. Positive controls consistently yielded PCR products of the expected sizes, and no amplification was observed in negative controls. The mPCR conditions were 95°C for 5 min followed by 28 cycles of 95°C for 30 s, 62°C for 30 s, 72°C for 30 s, and a final extension at 72°C for 5 min.

An additional mPCR targeting *stx1* and *stx2* was used to screen for Enterohemorrhagic *E. coli*. This assay was performed only on isolates in which the *eae* gene was detected. In the present study, this corresponded to a single isolate. The sizes of the expected mPCR products and the concentrations of the primer pairs are listed in Supporting Information S1: Table 1. The mPCR amplification was conducted in a total reaction volume of 25 μL containing 12.5 μL of Gotaq Green Master Mix 2X, 10.5 μL of Nuclease‐Free Water, 1 μL of DNA template, and the corresponding primers. Nuclease‐free water was used for the negative control. DNA from Enterohemorrhagic *E. coli* O157:H7 ATCC 43895 was used as a positive control. The mPCR conditions were 94°C for 5 min followed by 35 cycles of 94°C for 30 s, 62°C for 30 s, 72°C for 1 min, and a final extension at 72°C for 5 min.

### 2.3 Antibiotic Resistance Testing

PCR‐confirmed *E. coli* isolates were tested for susceptibility to 12 different antibiotics using the disk diffusion method on Mueller–Hinton Agar according to the guidelines of the Clinical Laboratory Standard Institute (CLSI, 2023). At least two isolates from each sample were tested. The antibiotics tested and the corresponding disk concentrations are summarized in Supporting Information S1: Table 2. Inhibition zone diameters around the antibiotic‐impregnated disks were measured in mm and rounded to the closest integer before being compared to the CLSI clinical break points to classify each bacterial isolate as resistant, intermediate, or susceptible. Multidrug resistance was defined as resistant to three or more different antibiotics. *E. coli* ATCC 25922 was used as a quality control strain.

### 2.4 DNA Extraction and Whole‐Genome Sequencing of Selected *E. coli* Isolates

DNA was extracted from selected *E. coli* isolates, including pathogenic *E. coli* and MDR *E. coli,* based on the antibiotic resistance phenotype using the Qiagen DNeasy Blood & Tissue Kit following the manufacturer′s instructions. Metadata for the eight isolates selected for WGS, including meat type, cut type, sampling month, and vendor, are provided in Supporting Information S1: Table 3. Genomic DNA extracts were prepared for sequencing using the Illumina DNA Prep kit (Illumina, San Diego, CA, USA) following the manufacturer's instructions. Whole‐genome sequencing was performed using the Illumina NextSeq. 1000 platform (Illumina, San Diego, CA, USA), generating paired‐end reads (2×150 bp).

### 2.5 Bioinformatics and Statistical Analyses

Sequencing data were subjected to filtering and trimming using fastp v0.23.4 (Chen et al. 2018) with the flags “detect_adapter_for_pe”, “trim_poly_g”, “cut_right”, “‐f 20”, and “‐F 20” activated. Respectively, these flags enable automatic detection and trimming of sequencing adapters, trim artifact poly‐G basecalls, trim downstream of read regions that fall below the quality threshold or show a poly‐G basecall, and trim the first 20 bases of forward and reverse reads. Successful trimming and read quality control were confirmed using FastQC v0.11.9 (Leggett et al. 2013). *De novo* genome assembly was performed using Unicycler v.0.5.0 (Wick et al. 2017) and default parameters. Genome assembly quality was assessed using CheckM, and all genomes showed high completeness and low contamination, supporting their suitability for downstream analyses (Supporting Information S1: Table 4).

Assembled genomes were annotated using the Bacterial and Viral Bioinformatics Resource Center (BV‐BRC) Genome Annotation Service, which integrates the RAST tool kit (RASTtk) for bacterial genome annotation (Brettin et al. 2015). Annotated genome data were used for phylogenetic analysis as described below.

For antibiotic resistance and virulence gene detection, assembled genome sequences in FASTA format were screened using ABRicate (Seemann 2016) on the Galaxy platform (The Galaxy Community et al. 2022). Screening for antibiotic resistance determinants and virulence‐associated genes was conducted against the Comprehensive Antibiotic Resistance Database (CARD) (McArthur et al. 2013) and the Virulence Factor Database (VFDB) (Chen 2004), respectively.

To assess plasmid‐encoded resistance and virulence determinants, plasmid contigs were extracted from genomes using the geNomad platform (Camargo et al. 2024). These plasmid contigs were then screened using ABRicate (Seemann 2016) against both CARD (McArthur et al. 2013) and the VFDB (Chen 2004) on the Galaxy platform (The Galaxy Community et al. 2022) to detect mobile antibiotic resistance and virulence genes located on plasmids. Conjugation‐associated genes were identified in silico from plasmid contigs predicted by geNomad. In silico serotype prediction was performed using SerotypeFinder v2.0 (Joensen et al. 2015) and predicted pathogenicity was assessed using PathogenFinder v1.1 (Cosentino et al. 2013), both accessed through the Center for Genomic Epidemiology (CGE) platform. Sequence types (STs) of *E. coli* isolates were determined using the Achtman MLST scheme implemented on the Galaxy platforms (The Galaxy Community et al. 2022).

MGEs were identified using PlasmidFinder v2.1.6 (Carattoli et al. 2014) and IntegronFinder v2.0.5 (Rocha 2022), both available through the Galaxy platform (The Galaxy Community et al. 2022). PlasmidFinder was used to identify plasmid replicon types, and IntegronFinder was used to detect integrons and associated gene cassettes.

Phylogenetic analysis was performed using the Codon Tree service available in the BV‐BRC (Odey et al. 2023).

A total of eight *E. coli* isolates from retail chicken and beef products in Botswana were included in the phylogenetic analysis described above. For each isolate, the two most closely related publicly available *E. coli* genomes were identified using the Similar Genome Finder tool in BV‐BRC, which applies Mash/MinHash distance estimation to identify near neighbors based on k‐mer similarity (Ondov et al. 2016). In total, 16 public genomes were included alongside the eight isolates from Botswana, resulting in a data set of 24 genomes. The Codon Tree pipeline selected 500 single‐copy orthologous protein‐coding genes conserved across all genomes, aligned these genes at the codon level, concatenated the alignments, and constructed a phylogenetic tree using the neighbor‐joining algorithm, with branch lengths calculated under the GTRCAT model. Branch support values were estimated from 1000 bootstrap replicates. Tree visualization and annotation were performed in iTOL (Letunic and Bork 2021), and phylogenetic relationships were further interpreted in the context of host source and sample origin.

## 3 Results

### 3.1 Prevalence of *E. coli* Isolated From Chicken Meat and Beef Samples Available for Retail Sale

*E. coli* was detected in 54.7% (63/109) of chicken‐product and beef samples collected from retail markets, confirmed by the presence of the *phoA* gene, a molecular marker commonly used for *E. coli* identification (Luo et al. 2023). Positive samples were evenly distributed between chicken (33/62, 53.2%) and beef (30/47 and 62.7%, Table 1, *p* > 0.05, Fisher's Exact Test). Among chicken cuts, *E. coli* was most prevalent in the intestines (65.0%), followed by feet (52.9%), mixed pieces (41.7%), and egg (100%, based on a single sample). In beef samples, ox tripe had the highest prevalence (66.7%), followed by ground beef mince (64.3%) and beef bones (50.0%). *E. coli* was isolated more frequently from ground beef compared to beef bones, though this difference was not statistically significant (*p* > 0.05). Although the sample source by vendor was recorded (Table 1), no statistically significant differences in *E. coli* detection rates were observed between vendors.

**Table 1:** Prevalence of *Escherichia coli* isolates in chicken and beef meat products by vendor and cut type.

Meat type	Vendor	Cut Type	Positive samples (*n*)	Total tested (*n*)	Prevalence (%)
**Chicken**	A	Feet	3	5	60.0
		Intestine	7	11	63.6
		Mixed Pieces	4	10	40.0
	B	Feet	6	12	50.0
		Intestine	6	9	66.7
		Mixed Pieces	6	14	42.9
		Egg	1	1	100.0
	**Total**		**33**	**62**	**53.2**
**Beef**	A	Ground Beef	9	14	64.3
		Ox Tripe	1	2	50.0
		Bones	1	2	50.0
	B	Ground Beef	18	28	64.3
		Ox Tripe	1	1	100.00
	**Total**		**30**	**47**	**62.7**

*Note:* The table presents the number and percentage of *E. coli*‐positive samples detected in various cuts of chicken and beef from two vendors. Prevalence was calculated as the proportion of positive samples out of the total number tested for each cut and vendor. Samples were collected monthly between January and December 2022.

### 3.2 Phenotypic Antibiotic Resistance of *E. coli* Isolated From Chicken and Beef

**Figure 1:**
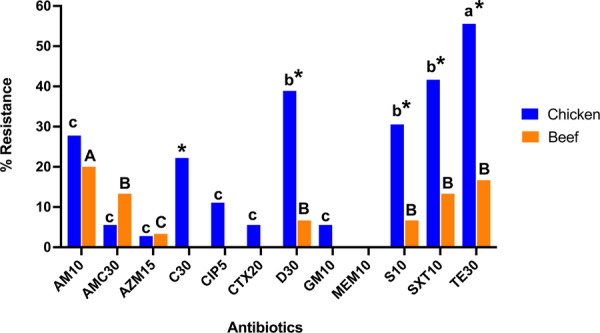
Percentage of *Escherichia coli* isolates classified as resistant to each antibiotic among isolates recovered from retail chicken (*n* = 33) and beef (*n* = 30) products. For each antibiotic, the percentage represents the proportion of resistant isolates out of the total number of isolates tested for the antibiotic within each meat type. Asterisks (*) indicate significantly higher % resistance in chicken isolates compared to beef isolates for the corresponding antibiotic (*p* < 0.05, Fisher's Exact Test). Comparisons of the % resistant to each antibiotic were performed within meat type, with significant differences indicated by different letters (a–c) above the bars. Capital letters are for beef and lowercase letters are for chicken. Bars sharing the same letter are not significantly different from each other, whereas bars with different letters represent significant differences (*p* < 0.05, Fisher's exact test). The abbreviations for antibiotics are as follows: ampicillin 10 μg (AM10), amoxicillin‐clavulanic acid 20/10 μg (AMC30), azithromycin 15 μg (AZM15), chloramphenicol 30 μg (C30), ciprofloxacin 5 μg (CIP5), cefotaxime 20 μg (CTX20), doxycycline 30 μg (D30), gentamicin 10 μg (GM10), meropenem 10 μg (MEM10), streptomycin 10 μg (S10), trimethoprim/sulfamethoxazole 10 μg (SXT10), and tetracycline 30 μg (TE30).

Of those meat samples contaminated with *E. coli* (*n* = 63), 55.6% (35/63) were resistant to at least one antibiotic. As shown in Figure 1, among chicken isolates, 69.7% (23/33) were resistant most commonly to tetracycline (54.5%, 18/33), trimethoprim/sulfamethoxazole (42.4%, 14/33), and doxycycline (39.4%, 13/33). Among isolates obtained from beef, 40.0% (12/30) demonstrated resistance most frequently to ampicillin (20.00%, 6/30), tetracycline (16.67%, 5/30), and trimethoprim/sulfamethoxazole (13.33%, 4/30, Figure 1). Meropenem resistance was not detected in any of the samples.

Multidrug resistance (MDR), defined as resistance to three or more antibiotics, was detected in 30.2% (19/63) of *E. coli* isolates obtained across meat products, with 45.5% (15/33) from chicken and 13.33% (4/30) from beef. Across chicken isolates, tetracycline resistance was significantly higher compared to resistance to other antibiotics (*p* < 0.05, Fisher's exact test). Ampicillin resistance in beef isolates was significantly more frequent than resistance to amoxicillin, azithromycin, chloramphenicol, ciprofloxacin, cefotaxime, and gentamicin (*p* < 0.05, Fisher′s Exact Test).

Comparisons between meat types revealed significantly higher resistance in chicken isolates than beef isolates for tetracycline, trimethoprim/sulfamethoxazole, doxycycline, streptomycin, and chloramphenicol (*p* < 0.05, Fisher's Exact Test), as shown in Figure 1. No significant differences were observed between chicken and beef isolates for resistance to ampicillin, ciprofloxacin, gentamicin, cefotaxime, amoxicillin/clavulanic acid, and azithromycin.

### 3.3 Whole‐Genome Sequencing of Selected *E. coli* Isolates

**Figure 2:**
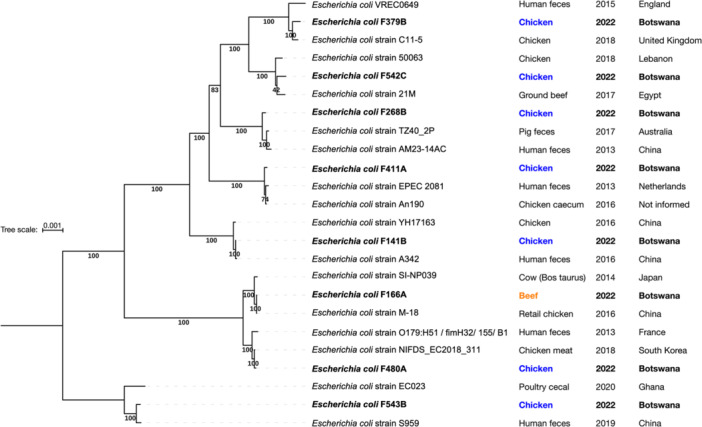
Neighbor‐joining phylogeny of *Escherichia coli* isolates from retail chicken and beef products collected in Botswana (shown in bold) and their closest publicly available genomes. The tree was constructed in BV‐BRC using neighbor‐joining with branch lengths calculated under the GTRCAT model and rooted on the longest branch, based on alignments of 500 single‐copy core genes identified through the BV‐BRC Codon Tree Service. The analysis included eight *E. coli* isolates from chicken (F141B, F268B, F379B, F411A, F480A, F542C, and F543B) and beef (F166A), along with 16 public *E. coli* genomes selected as the two closest neighbors for each isolate based on the Mash/MinHash distance. Host origin, year of isolation, and country of isolation are indicated in the label for each strain. The tree was visualized and annotated in iTOL (Interactive Tree of Life).

Whole‐genome phylogenetic analysis was conducted on eight *E. coli* isolates recovered from imported meat products (7 from chicken (F141B, F268B, F379B, F411A, F480A, F542C, and F543B) and 1 from beef (F166A)). Isolates were selected because they demonstrated resistance phenotypes to 3 or more antibiotics and were isolated during different months to better highlight potential *E. coli* diversity. All genomes from this study clustered among publicly available *E. coli* genomes deposited in the BV‐BRC database, with a high percentage similarity (> 99%). The sequenced isolates were dispersed across different branches of the phylogenetic tree (Figure 2), indicating high genomic diversity and the absence of any dominant lineage. Several chicken‐derived isolates clustered closely with publicly available *E. coli* genomes from chicken or human sources originating from multiple countries, including China, France, Ghana, and the United Kingdom, as shown in Figure 2, reflecting global genomic relatedness rather than direct trade‐linked origins.

### 3.4 *In silico* Multilocus Sequence Typing (MLST) Analysis

**Figure 3:**
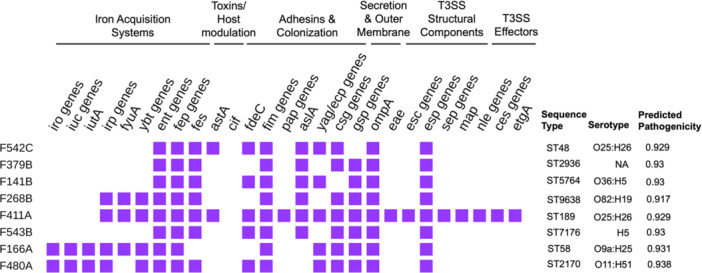
Virulence gene profiles, sequence types, serotypes, and predicted pathogenicity of *Escherichia coli* isolates from retail meat products. The heatmap displays the presence (purple squares) of key virulence‐associated genes in eight *E. coli* isolates using whole‐genome sequence data. *In silico* multilocus sequence typing (MLST) identified diverse sequence types of each isolate. Serotypes and predicted pathogenicity scores were identified using SerotypeFinder and PathogenFinder, respectively. A detailed list of virulence genes and their genomic locations is presented in Supporting Information S1: Table 5.

*In silico* MLST based on the Achtman MLST scheme using the WGS data of the eight sequenced *E. coli* isolates from chicken and beef identified a total of eight distinct sequence types (STs) (Figure 3). Seven chicken isolates showed unique STs, including ST5764, ST48, ST2170, ST2936, ST189, ST9638, and ST7176. One beef isolate displayed ST58.

### 3.5 *In Silico* Serotype Characterization of *E. coli* Isolates

Serotype prediction revealed diverse O:H combinations among the eight sequenced *E. coli* isolates (Figure 3). F543B was identified only by the H antigen (H5), likely due to the absence or divergence of O antigen gene clusters. F379B could not be assigned a serotype, possibly due to incomplete genome assembly or the presence of a novel or uncharacterized serotype.

### 3.6 Virulence Gene Profiles and Predicted Pathogenicity of *E. coli* Isolates

The presence of virulence‐associated genes in *E. coli* isolates from chicken and beef samples was assessed to evaluate their potential pathogenicity to humans, while also considering their relevance to animal hosts, given the zoonotic nature of several *E. coli* pathotypes. All eight *E. coli* isolates harbored multiple virulence‐associated genes, with detected virulence factors and their genomic locations summarized in Figure 3 and Supporting Information S1: Table 5. Virulence genes not detected in this study are not listed. Although the specific gene profiles varied among isolates, all were predicted by PathogenFinder to harbor virulence‐associated genomic features indicative of potential human pathogenicity, with probability scores above 0.9, suggesting that they could represent intestinal or extraintestinal pathotypes and highlighting the potential for these zoonotic isolates to be carried or result in illness in different hosts, particularly under conditions of compromised food hygiene or increased host susceptibility. F411A may be classified as an atypical EPEC, as it possessed *eae*, other genes in the LEE island, and fimbrial genes characteristic of EPEC isolates, but did not possess *bfp*, a gene encoding bundle‐forming pili typically associated with typical EPEC strains. Major gene clusters associated with virulence, including adherence genes (*csg, pap, fim,* and *afa*) and iron‐uptake genes (*fyuA*, *ybt*, *irp, iucA‐D*, *iutA*, *iroB‐E*, and *N*), which enhance bacterial survival and growth in iron‐limited host environments, were present. Notable virulence features were identified in other isolates. For instance, F542C and F411A carried the *astA* gene encoding a heat‐stable enterotoxin. Type II secretion system genes (*gspC‐M*) were present in most isolates, enabling the secretion of toxins and enzymes that facilitate host invasion. Together, virulence genes were detected on both chromosomal and plasmid contigs across the eight *E. coli* isolates from imported meat products.

### 3.7 Antibiotic Resistance Genes

**Figure 4:**
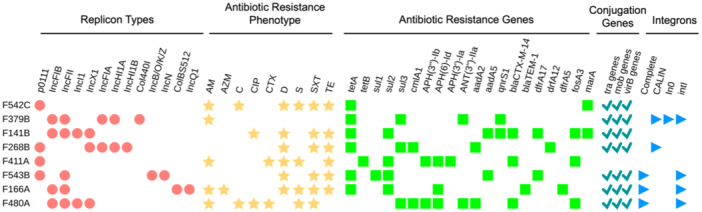
Whole‐genome sequence (WGS)–based in‐depth genetic characterization of eight multidrug‐resistant *Escherichia coli* isolates from retail meat, showing plasmid replicon types, antibiotic resistance (AR) phenotype, antibiotic resistance genes, and mobile genetic elements. The heatmap summarizes the presence of plasmid types (red circles), AR phenotypes (yellow stars), AR genes (light green squares), conjugation genes (green checkmarks), and integrons (blue triangles) in eight *E. coli* isolates from retail meat. The abbreviations for antibiotics are as follows: ampicillin (AM), azithromycin (AZM), chloramphenicol ©, ciprofloxacin (CIP), cefotaxime (CTX), doxycycline (D), streptomycin (S), trimethoprim/sulfamethoxazole (SXT), and tetracycline (TE). A detailed list of antibiotic resistance genes, conjugation‐associated genes, and their genomic locations is presented in Supporting Information S1: Table 6.

Figure 4 summarizes the distribution of AR genes identified across the eight *E. coli* genome assemblies from chicken and beef samples through annotation against the CARD database. AR genes were detected on both plasmid and chromosomal contigs across all *E. coli* isolates (Figure 4 and Supporting Information S1: Table 6). A diverse set of plasmid‐associated AR genes was identified, including clinically important resistance determinants conferring resistance to *β*‐lactams, aminoglycosides, quinolones, tetracyclines, and sulfonamides. Notably, the extended‐spectrum *β‐*lactamase (ESBL) gene *bla*_CTX‐M‐14_ was found on plasmids in isolates F411A, F141B, and F480A. The *bla*_TEM‐1_ gene was present on a plasmidic contig in F166A, but on a chromosomal contig in F480A. Quinolone resistance was supported by the detection of *qnrS1* in F141B and F379B, whereas sulfonamide resistance genes (*sul1*, *sul2*, or *sul3*) were widely distributed among plasmid contigs in six isolates. Tetracycline resistance genes (*tetA* and *tetB*) and aminoglycoside‐modifying enzyme genes (*aadA2*, *aadA5*, *APH(3”)‐Ib*, *APH(6)‐Id*, and *ANT(3”)‐IIa*) were also commonly detected on plasmids. Additionally, trimethoprim resistance genes from the dfrA family (*dfrA1*, *dfrA5*, *dfrA12*, *dfrA14*, and *dfrA17*) were identified on plasmid contigs in multiple isolates.

Conjugation‐associated genes, including several *tra* and *virB* operons, and *mob* genes, were identified in all isolates, except for F411A, suggesting that most plasmid‐borne AR genes have the potential to be horizontally transferred. Among the isolates, F543B harbored a more extensive repertoire of conjugation‐associated genes, including multiple *tra* genes and components of the type IV secretion system, compared to other isolates (Supporting Information S1: Table 6). These findings indicate that retail meat isolates may carry MGEs capable of disseminating multidrug resistance traits.

Chromosomally encoded resistance genes were more consistent across isolates and primarily included efflux systems (*acrAB*, *mdtABC*, *emrAB*, and *tolC*), global regulators (*marA*, *CRP*, and *H‐NS*), and membrane‐associated proteins (*mdfA*, *yojI*, and *bacA*). The *ampC* gene, encoding a chromosomal class C *β‐*lactamase, was present in all isolates.

### 3.8 Mobile Genetic Elements

A total of 13 distinct plasmid replicon types were identified *in silico* across the eight sequenced isolates, with IncF‐type plasmids being the most frequently detected. Among the chicken isolates, IncFIB(AP001918) and IncFII were the most prevalent, present in six out of seven isolates. Additionally, p0111, IncX1, and IncI1 plasmids were detected in multiple chicken isolates.

F141B, a chicken isolate, showed the most diverse plasmid profile, harboring IncFIB(AP001918), IncFII, IncI1, and IncX1 replicon types. Notably, F543B was the only isolate to carry IncB/O/K/Z and IncN replicons.

In contrast, the single beef isolate (F166A) showed less diversity in its plasmid profiles, harboring Col(BS512), IncFIB, IncFII, and IncQ1 replicons, suggesting a limited plasmid‐associated resistance profile compared to chicken‐product isolates.

Integron detection revealed that five out of eight isolates carried integrons, with variations in structural types and *attC* sites. Among the chicken isolates, F268B and F379B harbored CALIN (Clustered *attC* sites lacking integron integrase)‐type integrons, characterized by multiple *attC* sites but lacking an integrase gene, suggesting remnants of historical cassette recombination events. Notably, F379B also contained an In0‐type integron, which included the *intI* gene without associated *attC* sites, indicating a potentially incomplete integron. Three isolates (F166A, F480A, and F543B) possessed complete integrons containing both the integrase gene and one or more *attC* sites, with F480A showing the most extensive structure, including five *attC* sites and multiple putative cassette regions. In contrast, isolates F411A, F141B, and F542C did not show evidence of any integron elements.

## 4 Discussion

The global rise of antibiotic resistance is occurring alongside an increasing incidence of *E. coli*‐associated diseases (Kaper et al. 2004). These include diarrheal illnesses caused by intestinal pathotypes (IPEC) such as EPEC and EHEC. In addition, invasive extraintestinal infections, such as those found in urinary tract infections and sepsis, are also on the rise, caused by ExPEC, UPEC, and APEC (Sarowska et al. 2019). Industrialized nations report higher incidences of AR (Van Boeckel et al. 2019; WHO 2021a). Meat is a well‐recognized reservoir of IPEC, and more recently, chicken has also been implicated as a source of ExPEC strains that colonize the gut and contribute to infections at extraintestinal sites under certain host and exposure conditions. This underscores meat as a reservoir of *E. coli* with diverse pathogenic potential, rather than as an inherent predictor of disease outcome (Ingle et al. 2018; Malesa et al. 2024). In regions with limited local agricultural production, such as northern Botswana, retail meat products are largely imported to meet consumer demand. Here, imported meat refers to retail meat products introduced into the Chobe region from production systems outside the region. Within this context, imported meat may represent a potential pathway for the introduction of antibiotic‐resistant *E. coli* into new ecological settings. Although environmental dissemination was not directly assessed in this study, indirect pathways such as food handling practices, human waste streams, and subsequent contamination of water or soil could theoretically contribute to wider spread. This issue is particularly relevant in regions like Botswana, where imports US$17.77 million (2023) of meat, poultry, and seafood are high to meet demand, but where monitoring and systematic surveillance of antibiotic‐resistant *E. coli* in imported foods and human populations remain limited. (Botswana Imports of Meat fish and seafood preparations ‐ 2025 Data 2026 Forecast 2000–2023 Historical). It is also important to acknowledge that international travel, environmental exposure, and other transmission routes may contribute to the introduction of antibiotic‐resistant bacteria and should be considered alongside foodborne pathways. WGS of antibiotic‐resistant *E. coli* isolates from chicken and beef collected in 2022 identified a diversity of genes conferring resistance to antibiotics commonly used for treatment, and MGEs that may contribute to the spread of AR to other bacteria in the region. Although only eight isolates were sequenced, it is likely that some of the ARGs identified are also present in other *E. coli* from retail meats, as 55.6% of isolates from retail meat products showed resistance to at least one antibiotic and 30.2% were multidrug‐resistant (MDR), with a higher proportion in chicken‐meat isolates (45.5%) compared to beef (13.3%). The predominance of resistance to tetracycline, trimethoprim/sulfamethoxazole, and doxycycline in chicken‐meat isolates likely reflects the widespread and often unregulated use of these antibiotics in commercial poultry farming (Tepeli and Demirel Zorba 2018; Sharma et al. 2022). These regional patterns align with the resistance profiles observed in chicken‐derived isolates in this manuscript, particularly the elevated resistance to tetracyclines and trimethoprim/sulfamethoxazole shown in Figure 1. These findings are consistent with previous reports from poultry production systems in southern Africa, a region that includes key food trade partners to Botswana. Surveillance studies from South Africa have documented high levels of antibiotic resistance in poultry‐associated *E. coli*, particularly to tetracyclines and sulfonamides, with resistance to tetracycline exceeding 70%–80% across multiple sampling years, and approximately 80% of poultry‐derived isolates were classified as MDR (Theobald et al. 2019). Although resistance levels observed in the present study were relatively lower, chicken isolates similarly showed a predominance of resistance to tetracycline and trimethoprim/sulfamethoxazole, and a substantially higher proportion of MDR isolates compared to beef, suggesting shared selective pressures within regional poultry production systems. The presence of MDR *E. coli* in meat raises significant concerns regarding potential human exposure, particularly through improper handling and cross‐contamination during food preparation, even in the absence of confirmed pathogenicity or demonstrated plasmid transfer. Such exposure may facilitate transient colonization or the dissemination of resistance genes under favorable conditions (Sharma et al. 2018). In this context, educating food handlers on safe handling and preparation practices, including measures to prevent cross‐contamination, represents a critical and immediately actionable intervention to reduce foodborne transmission of antibiotic‐resistant bacteria.

WGS of eight MDR isolates revealed diverse profiles of AR genes and virulence factors. Importantly, although some resistance phenotypes were consistent with the presence of specific resistance genes, discrepancies were observed in the case of quinolone resistance. For example, although only F480A showed phenotypic resistance to ciprofloxacin (CIP), *qnrS1* was detected in two other isolates (F141B and F379B), all of which displayed intermediate susceptibility to CIP. In contrast, F480A, which lacked *qnr*S1, carried a *gyrA* mutation that likely explains its high‐level resistance to CIP, highlighting the role of chromosomal mutations in fluoroquinolone resistance, especially when plasmid‐mediated resistance is absent.

Interestingly, the eight sequenced isolates showed substantial genetic and phylogenetic diversity. Distinct STs and serotypes were predicted in silico for each isolate, with no evidence of a dominant clonal lineage. This diversity contrasts with the phenotypic AR resistance similarities and indicates that multiple MDR *E. coli* strains circulating in meat have acquired comparable resistance profiles through horizontal gene transfer. Phylogenetic analysis further confirmed the genomic heterogeneity, as the isolates clustered into distinct branches of the core genome tree and frequently grouped with chicken‐ and human‐associated *E. coli* strains from other countries, including China, France, Ghana, and Lebanon. Although these countries do not represent the likely origins of retail meat products in Botswana, their inclusion provides comparative genomic context and highlights the global circulation of diverse *E. coli* lineages across food‐ and human‐associated systems. Rather than implicating direct trade links, the observed relatedness may instead reflect shared inputs in poultry production, including feed ingredients that may be imported or shared across regions (Da Costa et al. 2007; Tsai et al. 2025). Poultry feed has been recognized as a vehicle for ARB and resistance genes that can colonize the gut microbiota and promote horizontal gene transfer (HGT) under antibiotic pressure (Da Costa et al. 2007; De Mesquita Souza Saraiva et al. 2022). Contaminated feed may thus contribute to the emergence and persistence of MDR *E. coli* in poultry populations, supporting the need to consider upstream factors like feed sourcing in AR surveillance and control strategies (Da Costa et al. 2007).

The detection of plasmid‐encoded conjugation systems, integrons, and various resistance determinants across the majority of isolates suggests that MGEs may have played a key role in the acquisition of AR genes across the sequenced *E. coli*. Notably, all isolates except F411A carried conjugation‐associated genes, suggesting that HGT via conjugation could represent a possible mechanism for the dissemination of resistance genes. F543B, in particular, harbored a nearly complete conjugative machinery, including a full complement of *tra* and *trb* genes, mobilization (*mob*) genes, and Type IV secretion system (*virB*) components, suggesting the potential presence of a self‐transmissible plasmid. The co‐occurrence of multiple distinct conjugation gene sets in F543B also raises the possibility that this isolate may harbor more than one conjugative or mobilization plasmid, which may potentially enhance its ability to disseminate resistance genes. This isolate also carried IncB/O/K/Z and IncN replicons, both of which are typically involved in the transfer of resistance genes across bacterial species (Algarni et al. 2023; Yu et al. 2024). Seven isolates had IncF‐type plasmids, which are well‐documented vectors of multidrug resistance and virulence in *E. coli*, especially in strains from food and clinical sources (Carattoli 2009). These plasmids have been shown to frequently co‐localize resistance and virulence determinants, which may increase the fitness and transmission potential of host bacteria (Carattoli 2009; Johnson and Nolan 2009). The most diverse types of IncF‐type plasmids (IncFIA(HI1), IncFIB, and IncFII) were detected in F379B, indicating the presence of large, conjugative plasmids that are often associated with the dissemination of multidrug resistance genes (Yang et al. 2015; Chen et al. 2024).

The integrons detected in several isolates varied in structural completeness, including classic integrons with integrase and *attC* sites as well as incomplete forms such as CALINs and In0 types. The presence of these distinct integron architectures likely reflects ongoing and historical recombination activity and suggests a role for integrons in the accumulation and mobilization of AR genes (Stokes and Gillings 2011; Gillings 2014). In general, the co‐occurrence of integrons and conjugative plasmids has been recognized as exerting the potential synergistic effects of MGEs that may facilitate the spread of resistance traits across bacterial populations (Partridge et al. 2018; Che et al. 2021). In the present study, several isolates harbored integrons alongside plasmid‐associated resistance genes, a pattern that is consistent with this broader framework, although functional horizontal transfer was not directly assessed and some conjugation systems were incomplete. The presence of ESBL genes such as *bla*_CTX‐M‐14_, and *bla*_TEM‐1_, as well as other clinically important determinants, including *ampC*, *qnrS1*, *sul*, *aadA,* and *dfrA* variants, in all isolates, demonstrates the wide resistance present in food‐derived *E. coli*. Notably, several of these genes were located on plasmid contigs, supporting the possibility that plasmids contribute to the mobilization of resistance genes. These findings align with reports showing the dominance of IncF and class 1 integrons in MDR *E. coli* from animal and food sources (Yang et al. 2015; Rozwandowicz et al. 2018; Zhang et al. 2020). Furthermore, the detection of resistance determinants commonly reported in human clinical *E. coli* isolates within foodborne strains indicates a potential overlap between resistance gene pools in food‐ and human‐associated bacteria. Although direct genomic comparison with human clinical isolates from Botswana was not performed in this study, similar resistance determinants have been widely reported in clinical isolates globally, supporting the relevance of meat as a possible vehicle for ARB with potential to disseminate into the human population (Manges et al. 2019; WHO 2021b).

In addition to AR, the sequenced isolates possessed a wide array of virulence genes associated with multiple pathotypes. All eight isolates were predicted to be potential human pathogens by PathogenFinder, highlighting their clinical relevance and the potential risk posed by meat products. Most isolates harbored adhesin genes involved in intestinal colonization, including the *fim* operon (type 1 fimbriae), *fdeC*, *ecp* (*E. coli* common pilus), and *ompA*, supporting their potential to adhere to intestinal surfaces. F411A contains genes that are hallmarks of EPEC‐mediated diarrheal illness including the *eae* gene, a complete locus of the enterocyte effacement (LEE) pathogenicity island, and genes encoding the type III secretion system and associated effotors enabling close adherence to host intestinal epithelial cells (Gaytán et al. 2016; Braverman et al. 2023). Although *eae* was not present in the remaining eight isolates, several carried LEE‐encoded effector genes such as *espL1*, *espL4*, *espX1‐X5*, and *nleA* genes, many of which showed high sequence identity (> 96%) to homologs found in *E. coli* O157:H7. Although a complete LEE island was not detected in these isolates, the presence of multiple effectors with strong homology to LEE‐positive strains suggests horizontal acquisition of genes important in attachment and effacement. This pattern is consistent with previously reported hybrid or emerging pathotypes that carry virulence elements from multiple lineages (Hazen et al. 2017). In the absence of functional assays, these observations do not confirm pathogenic potential but raise the possibility of alternative subclinical virulence mechanisms resembling atypical EPEC strains (Furniss and Clements 2018). Such strains may be of particular concerns in populations with a high prevalence of immunosuppressive conditions, including HIV, where host susceptibility may increase the likelihood of colonization or progression to disease (Kaper et al. 2004; Manges et al. 2019). Importantly, the detection of virulence‐associated genes should not be interpreted as evidence of strict pathogenicity, as many *E. coli* strains function as opportunistic pathogens whose ability to cause disease depends on host susceptibility and exposure context.

Beyond intestinal pathogenicity, most isolates carried virulence determinants identified in ExPEC strains, including UPEC‐ and APEC‐like strains. These included *pap* operon (P fimbriae), *fim* operon (type 1 fimbriae), outer membrane protein *ompA*, and multiple iron acquisition systems such as *iro*, *iuc*, *irp*, and *ybt*, which are important for survival in iron‐limited environments like the urinary tract and bloodstream (Kariyawasam et al. 2006). These virulence genes are frequently associated with UPEC strains that cause urinary tract infections and APEC strains implicated in colibacillosis in poultry (Kathayat et al. 2021). Although well‐known APEC markers like *ompT*, *hlyF*, and *vat* were not detected, the presence of these alternative APEC‐associated virulence genes, particularly in combination, indicates zoonotic potential and supports their classification as APEC‐ and UPEC‐like strains (Sattar et al. 2023). Importantly, many of these virulence genes were located on plasmid contigs in isolates that also carried conjugative elements, suggesting a risk of HGT to commensal or pathogenic strains within the gut microbiota. Their presence in food‐derived isolates is important, as it suggests that improper handling of contaminated meat or compromised kitchen hygiene could serve as a transmission route for bacteria capable of causing not only intestinal illness but also invasive extraintestinal diseases in susceptible hosts.

Although the presence of antibiotic‐resistant and virulence‐associated genes indicates a potential for harm, the real‐world risk of human illness is strongly shaped by food‐handling and hygiene behaviors. Research has consistently shown that improper handling of raw meat, especially poultry, can lead to extensive cross‐contamination of hands, kitchen tools, and food‐contact surfaces, creating multiple opportunities for bacteria to spread to foods that will not be cooked again (Kirchner et al. 2023). Such transfer events substantially increase the likelihood of exposure, even when the original levels of contamination on the meat are low (Iulietto 2020; Kirchner et al. 2022). Quantitative microbiological risk assessment further demonstrates that cross‐contamination during domestic food preparation can represent a major pathway for pathogen transmission, in some cases contributing more to consumer exposure than insufficient cooking alone (Iulietto 2020). In these situations, strains that might otherwise act as harmless or opportunistic organisms can establish intestinal colonization or cause illness, particularly among infants, older adults, and other vulnerable groups (Kaper et al. 2004; Manges et al. 2019). These risks are magnified in environments where food safety practices are inconsistent or where access to sanitation infrastructure is limited. Therefore, food hygiene is a critical contextual factor when interpreting the public health relevance of genomic findings from retail chicken or beef products, as poor hygiene behaviors can transform a manageable hazard into a significant exposure pathway.

The findings of this study have important implications for food safety management, genomic surveillance strategies, and public health interventions aimed at limiting the dissemination of antibiotic‐resistant bacteria through the food supply chain. Imported meat products may serve as a potential reservoir or source for antibiotic‐resistant and potentially pathogenic *E. coli*, facilitating the introduction of MDR bacteria into local microbial ecosystems through food handling, consumption, waste production, and subsequent environmental contamination and dissemination. The presence of plasmid‐encoded resistance genes and MGEs supports the potential for horizontal gene transfer, which may facilitate the persistence and spread of AR traits across microbial communities and ecosystems. These risks are especially critical in regions with limited local meat production and high reliance on imports. The observed genetic diversity and mobility of resistance elements underscore the need for continuous genomic surveillance and high‐resolution molecular tools to track the emergence and spread of AR across landscape types, including those with little or no agricultural production. This is important not only for protecting consumer safety but also for informing risk‐based monitoring, targeted interventions, and food safety policies aimed at reducing the introduction and dissemination of antibiotic‐resistant bacteria along the food supply chain.

These findings also align with a broader One Health framework, underscoring how imported meat products may serve as vehicles for the introduction of novel antibiotic‐resistant *E. coli* strains and associated genes into the local environment and animal communities, both wild and domesticated. The Chobe region of Botswana, characterized by its rich biodiversity and protected ecosystems, has previously been shown to harbor antibiotic‐resistant *E. coli* in wildlife species, likely reflecting environmental contamination and transmission across human, animal, and environmental interfaces (Jobbins and Alexander 2015). Furthermore, Sanderson et al. (Rozwandowicz et al. 2018) reported highly similar antibiotic resistance profiles among *E. coli* isolates from surface water, wildlife, and humans in the Chobe region, indicating potential transmission of ARB between environmental sources and the human population. In this manuscript, resistance phenotypes to amoxicillin and azithromycin in foodborne *E. coli* that have not previously been reported in regional wildlife were identified. Together, these findings suggest that imported meat may represent one of several potential pathways through which resistance genes associated with clinically important antibiotics are introduced into the local ecosystem, where they may persist and potentially spread to both wildlife and human populations. The Chobe region features a large proportion of land under conservation protection and supports dense and diverse wildlife populations, many of which interact with human settlements or water sources near urban areas. If ARB or resistance genes introduced through imported meat contaminate the environment, they may be taken up by wildlife species, particularly scavenging species. Although such bacteria do not pose a direct threat to wildlife health, wildlife may serve as secondary reservoirs or conduits for antibiotic resistance, facilitating transmission back to humans and increasing the risk of treatment failure (Allen et al. 2010). Given the high mobility of resistance elements observed in the isolates in this study, there is a risk of potential introduction of HGT to native microbial populations, further expanding the regional resistome. Together, these findings support the hypothesis that retail meat may represent a critical pathway for the introduction of both pathogenic and antibiotic‐resistant *E. coli* into novel systems, facilitating the amplification and dissemination of resistance through human waste deposited in ecologically sensitive and interconnected environments.

## 5 Conclusion

The results of this study highlight the potential for imported beef and chicken meat to act as a critical transmission route linking agriculturally dominated ecosystems, their pathogen communities, and antibiotic resistance with novel environments. Although beef and chicken are recognized as high‐risk sources of antibiotic‐resistant bacteria, their contribution to extra‐intestinal infections and associated morbidity and mortality remains underestimated. In Botswana, like many dryland countries where food imports are essential for food security, this risk is especially acute for vulnerable populations, including individuals with immunosuppressive conditions such as human immunodeficiency virus (HIV, Liu et al. 2025).

The global movement of agricultural goods accelerates the spread of AR from industrialized systems to remote regions, amplifying the threat to public health and environmental stability. This underscores the urgent need for expanded genomic surveillance across food, environmental, and clinical sources to track resistance emergence and dissemination. Public health agencies must prioritize screening of imported products and implement critical control points throughout beef and poultry supply chains. Additionally, educating food handlers on safe handling and preparation practices, including measures to prevent cross‐contamination, is essential to reducing the burden of foodborne illness.

## Author Contributions

**Saehah Yi:** writing – original draft, writing – review and editing, formal analysis, investigation, visualization. **Kathleen A. Alexander:** writing – review and editing, supervision, resources, project administration, methodology, funding acquisition, conceptualization. **Auja Bywater:** investigation, data curation. **Galaletsang Dintwe:** investigation, data curation. **Ashton N. Sies:** writing – review and editing, investigation, data curation. **Thomas H. Haidl:** formal analysis, data curation. **Andrew D.S. Cameron:** writing – review and editing, resources, funding acquisition, supervision. **Monica A. Ponder:** writing – original draft, writing – review and editing, resources, supervision, project administration, methodology, funding acquisition, data curation, conceptualization.

## Ethics Statement

The authors have nothing to report.

## Conflicts of Interest

Kathleen Alexander is the board president of CARACAL. CARACAL was a sub‐recipient on a grant that funded this work (NSF award #2009717).

## Supporting information


**Supplementary Table 1:** The E. coli primers used and their corresponding attributes. **Supplementary Table 2:** Antibiotics and disk concentrations used for phenotypic antibiotic susceptibility testing for Escherichia coli isolates. **Supplementary Table 3:** Metadata for Escherichia coli isolates selected for whole‐genome sequencing, including meat type, cut type, sampling month, and vendor. **Supplementary Table 4:** Genome assembly and quality metrics of sequenced Escherichia coli isolates. **Supplementary Table 5:** Genomic distribution of virulence genes among Escherichia coli isolates from retail meat products. **Supplementary Table 6:** Distribution of antibiotic resistance genes and conjugation‐associated elements in Escherichia coli isolates from retail meat products.

## Data Availability

The data sets generated and analyzed during the current study are available in the NCBI Sequence Read Archive (SRA) under BioProject accession PRJNA1306635 at https://www.ncbi.nlm.nih.gov/bioproject/PRJNA1306635.
