# Supplementary material for: Imported Retail Beef and Chicken Meat Products Serve as Reservoirs for Emerging Antibiotic‐Resistant Pathotypes of Escherichia coli in Pristine Areas Free From Agricultural Activity
Source: Microbiologyopen. 2026 Mar 30;15(2):e70273. doi: 10.1002/mbo3.70273 (PMC13140742; doi:10.1002/mbo3.70273)
Supplement: Supplementary file 1 — Supplementary Table 1: The E. coli primers used and their corresponding attributes. Supplementary Table 2: Antibiotics and disk concentrations used for phenotypic antibiotic susceptibility testing for Escherichia coli isolates. Supplementary Table 3: Metadata for Escherichia coli isolates selected for whole‐genome sequencing, including meat type, cut type, sampling month, and vendor. Supplementary Table 4: Genome assembly and quality metrics of sequenced Escherichia coli isolates. Supplementary Table 5: Genomic distribution of virulence genes among Escherichia coli isolates from retail meat products. Supplementary Table 6: Distribution of antibiotic resistance genes and conjugation‐associated elements in Escherichia coli isolates from retail meat products. [file MBO3-15-e70273-s001.docx]

**Supplementary Table 1:** The *E. coli* primers used and their corresponding attributes.

| **Gene** | **Sequence (5’ to 3’)** | **Size (bp)** | **Target pathotype** | **Concentration** | **Reference** |
| --- | --- | --- | --- | --- | --- |
| *eae - F* | TCAATGCAGTTCCGTTATCAGTT | 482 | *EHEC* | 0.4 μM | (Vidal et al., 2005) |
| *eae - R* | GTAAAGTCCGTTACCCCAACCTG |  |  | 0.4 μM |  |
| *phoA - F* | GTGACAAAAGCCCGGACACCA TAAATGCCT | 903 | *E. coli control* | 0.24 μM | (Kong et al., 1999) |
| *phoA - R* | TACACTGTCATTACGTTGCGGATTTGGCGT |  | *E. coli control* | 0.24 μM |  |
| *est1b - F* | TGTCTTTTTCACCTTTCGCTC | 171 | *ETEC* | 0.2 μM | (Chandra et al., 2013) |
| *est1b - R* | CGGTACAAGCAGGATTACAACAC |  |  | 0.2 μM |  |
| *stx1 - F* | CGATGTTACCGTTTGTTACTGTCACAG | 216 | *EHEC* | 0.4 μM | (Müller et al., 2007) |
| *stx1 - R* | AATGCCACGCTTCCCAGAATT |  |  | 0.4 μM |  |
| *stx2 - F* | GTTTTGACCATCTTCGTCTGATTATTGAG | 343 | *EHEC* | 0.4 μM | (Müller et al., 2007) |
| *stx2 - R* | AGCGTAAGGCTTCTGCTGTGAC |  |  | 0.4 μM |  |

**Supplementary Table 2.** Antibiotics and disk concentrations used for phenotypic antibiotic susceptibility testing for *Escherichia coli* isolates.

| Antibiotic | Abbreviation | Disk Concentration (μg ) |
| --- | --- | --- |
| Amoxicillin-clavulanic acid | AMC | 20/10 |
| Ampicillin | AM | 10 |
| Azithromycin | AZM | 15 |
| Chloramphenicol | C | 30 |
| Cefotaxime | CTX | 20 |
| Ciprofloxacin | CIP | 5 |
| Doxycline | D | 30 |
| Gentamicin | GM | 10 |
| Meropenem | MEM | 10 |
| Streptomycin | S | 10 |
| Tetracycline | TE | 30 |
| Trimethoprim/ sulfamethoxazole | SXT | 10 |

**Supplementary Table 3.** Metadata for *Escherichia coli* isolates selected for whole-genome sequencing, including meat type, cut type, sampling month, and vendor. The isolates were selected based on antibiotic resistance phenotypes and were used for comparative genomic and phylogenetic analyses.

| Isolate ID | Meat Type | Cut Type | Sampling Month | Vendor |
| --- | --- | --- | --- | --- |
| F411A | Chicken | Intestine | Aug-22 | A |
| F141B | Chicken | Mixed pieces | Feb-22 | A |
| F268B | Chicken | Intestine | May-22 | A |
| F542C | Chicken | Feet | Nov-22 | B |
| F379B | Chicken | Intestine | Jul-22 | A |
| F543B | Chicken | Feet | Nov-22 | A |
| F480A | Chicken | Intestine | Sep-22 | A |
| F166A | Beef | Ground Beef | Feb-22 | A |

**Supplementary Table 4.** Genome assembly and quality metrics of sequenced *Escherichia coli* isolates.

| Isolate ID | Meat Type | Contigs (n) | Genome length (bp) | GC content (%) | Contig N50 (bp) | Contig L50 | CheckM completeness (%) | Coarse consistency (%) | Fine consistency (%) |
| --- | --- | --- | --- | --- | --- | --- | --- | --- | --- |
| F411A | Chicken | 412 | 5,261,470 | 50.42 | 89,684 | 20 | 100 | 99.7 | 98.5 |
| F141B | Chicken | 151 | 4,890,503 | 50.77 | 115,807 | 13 | 100 | 99.9 | 98.7 |
| F268B | Chicken | 192 | 4,810,662 | 50.75 | 129,402 | 12 | 100 | 99.7 | 98.5 |
| F542C | Chicken | 262 | 4,904,524 | 50.63 | 123,021 | 13 | 100 | 99.9 | 98.8 |
| F379B | Chicken | 257 | 4,962,596 | 50.5 | 92,956 | 17 | 100 | 99.7 | 98.7 |
| F543B | Chicken | 321 | 5,042,698 | 50.72 | 60,895 | 23 | 100 | 99.4 | 96.8 |
| F480A | Chicken | 215 | 5,159,639 | 50.61 | 225,185 | 8 | 100 | 99.7 | 98.7 |
| F166A | Beef | 162 | 4,903,738 | 50.75 | 155,307 | 11 | 100 | 99.9 | 99.3 |

**Supplementary Table 5.** Genomic distribution of virulence genes among *Escherichia coli* isolates from retail meat products.

| **Isolate** | **Source** | **Plasmid-Associated Virulence Genes** | **Chromosome-Associated Virulence Genes** |
| --- | --- | --- | --- |
| F411A | Chicken | *astA, cesAB, cesD, cesD2, cesF, cesL, cesT, eae, escE, escL, escR, escS, escT, escU, escC, escJ, escI, escV, escN, escO, escP, escD, escF, escG, espR1, espG, espH, espA, espD, espF, etgA, fdeC, fimA, fimB, fimC, fimD, fimE, fimF, fimG, fimH, fimI, map, sepD, sepQ/escQ, sepL, papB, papI, papC, papD, papE, papF, papG, papH, papJ, papK, yagV/ecpE, yagW/ecpD, yagX/ecpC, yagY/ecpB, yagZ/ecpA, ykgK/ecpR* | *aslA, cif, csgG, csgF, csgD, csgB, entD, entF, entS, entC, entE, entB, entA, espJ, espL1, espM2, espX5, espX4, espR1, espG, escE, fepA, fepB, fepC, fepG, fepD, fes, fyuA, gspC, gspD, gspE, gspF, gspG, gspH, gspI, gspJ, gspK, gspL, gspM, irp1, nleA/espI, nleB2, nleH1, nleA, ompA, ybtU, ybtT, ybtE, ybtS, ybtX, ybtQ, ybtP, ybtA* |
| F141B | Chicken | *espR1, fdeC, fimA, fimE, fimB, fimI, fimC, fimD, fimF, fimG, fimH, yagV/ecpE, yagW/ecpD, yagX/ecpB, yagZ/ecpA, ykgK/ecpR* | *aslA, csgB, csgD, csgF, csgG, espY1, espX1, espX4, espX5, espL1, entA, entB, entC, entD, entE, entF, entS, fepA, fepB, fepD, fepG, fepC, fes, gspC, gspD, gspE, gspF, gspG, gspH, gspI, gspJ, gspK, gspL, gspM, ompA,* |
| F268B | Chicken | *espR1* | *aslA, csgB, csgD, csgF, csgG, entA, entB, entD, entE, entC, entS, entF, espL1, espX1, espX4, espX5, espY1, fepA, fepB, fepD, fepG, fepC, fes, fimH, fimG, fyuA, gspC, gspH, gspI, gspJ, gspK, gspL, gspM, gtrA, irp2, irp1, ompA, ybtE, ybtU, ybtT, ybtX, ybtQ, ybtP, ybtA* |
| F542C | Chicken | *astA, entA, entB, entE, entC, entS, entD, espR1, fdeC, fepB, fepD, fepG, fepC, fepA, fes, fimH, fimG, fimF, fimD, fimC, fimI, fimA, fimE, fimB, ykgK/ecpR, yagZ/ecpA, yagX/ecpC, yagW/ecpD, yagV/ecpE* | *aslA, csgG, csgD, csgF, csgB, entF, espL1, espL4, espX1, espX4, espX5, espY1, ompA* |
| F379B | Chicken | *fimA, fimB, fimC, fimD, fimE, fimF, fimG, fimH, fimI* | *aslA, csgG, gspF, csgD, csgB, entA, entB, entE, entC, entD, entS, entF, espL1, espL4, espX1, espX4, espX5, fepA, fepB, fepG, fepC, fes, gspL, gspM, gspK, gspJ, gspH, gspG, gspF, gspE, gspD, ompA* |
| F543B | Chicken | *csgB, csgD, csgF, csgF, csgG, fdeC, fimB, fimE, fimA, fimI, fimC, fimD, fimF, fimG, fimH* | *aslA, entA, entB, entE, entC, entF, entS, espR1, espX1, espX2, espX4, espX5, espL1, espL4, espY4, fdeC, fepA, fepB, fepD, fepG, fepC, fes, gspD, gspE, gspF, gspG, gspH, gspI, gspJ, gspK, gspL, ompA* |
| F480A | Chicken | *fdeC, iroN, iroE, iroD, iroC, iroB, iucA, iucB, iucC, iucD, iutA* | *csgB, csgD, csgF, csgG, entA, entB, entC, entD, entE, entF, entS, espX1, espX4, espX5, espL1, espR1, fepA, fepB, fepC, fepD, fepG, fes, fimH, fimG, fimF, fimD, fimC, fimI, fimA, fimE, fimB, fyuA, gspC, gspD, gspE, gspF, gspG, gspH, gspI, gspJ, gspK, gspL, gspM, irp1, irp2, ompA, yagV/ecpE, yagW/ecpD, yagX/ecpC, yagY/ecpB, yagZ/ecpA, ykgK/ecpR, ybtE, ybtT, ybtU, ybtA, ybtP, ybtQ, ybtY, ybtS* |
| F166A | Beef | *espR1, fdeC, iroB, iroC, iroD, iroE, iroN, iucD, iucC, iucB, iucA, iutA, ykgK/ecpR, yagZ/ecpA, yagY/ecpB, yagX/ecpC, yagW/ecpD* | *csgB, csgD, csgF, csgG, entA, entB, entC, entD, entE, entF, entS, espL1, espX1, espX4, espX5, fepA, fepB, fepC, fepG, fepD,fimH, fimG, fimF, fimD, fimC, fimI, fimA, fimE, fimB, fyuA, gspM, gspL, gspK, gspJ, gspI, gspH, gspG, gspE, gspD, gspC, irp1, irp2, ompA, ybtS, ybtX, ybtQ, ybtP, ybtA, ybtU, ybtT, ybtE,* |

**Supplementary Table 6.** Distribution of antibiotic resistance genes and conjugation-associated elements in *Escherichia coli* isolates from retail meat products.

| **Isolate** | **Source** | **Plasmid-associated Conjugation Genes** | **Plasmid-associated AR genes** | **Chromosome-associated AR genes** |
| --- | --- | --- | --- | --- |
| F411A | Chicken | none | *blaCTX-M-14, fosA3, eptA, tetB, sul2, APH(3'')-Ib, APH(6)-Id, APH(3')-Ia* | *cpxA, kdpE, emrR, emrA, emrB, ugd, mdtA, mdtB, mdtC, baeS, baeR, mphB, acrD, ampC (class C β-lactamase), msbA, mdtG, mdtH, H-NS, mdtN, mdtO, mdtP, ampH, acrB, acrA, yojI, mdtM, acrS, acrE, acrF, mdtE, mdtF, gadW, gadX, mdtA, marA, CRP, pmrF, emrY, emrK, evgA, evgS, tolC, bacA* |
| F141B | Chicken | *f_trae, f_traf, f_trag, f_trah, f_trak, f_tral, f_tran, f_trau, f_trav, f_traw, i_trae, i_trai, i_trak, i_tral, i_tran, i_trao, i_trap, i_traq, i_trar, i_trat, i_trav, i_traw, i_tray, i_trba, i_trbb, mobf, mobp1, t_virb9, trau, virb4* | *marA, eptA, mdtM, mdtN, mdtO, mdtP, qnrS1, sul2, aadA5, dfrA17, fosA3, blaCTX-M-14, tetA, mdtE, mdtF gadW, gadX* | *pmrF, yojI, baeR, baeS, mdtC, mdtB, mdtA, ugd, acrA, acrB, msbA, emrE, mdtH, mdtG, tolC, emrR, emrA, emrB, cpxA, kdpE, acrF, acrE, acrS, H-NS, CRP, bacA, mphB, acrD, ampC (class C β-lactamase), evgS, evgA, emrK, emrY, mdfA* |
| F268B | Chicken | *f_trae, f_traf, f_trag, f_trah, f_trak, f_tral, f_tran, f_trau, f_trav, f_traw, i_trae, i_trai, i_trak, i_tral, i_tran, i_trao, i_trap, i_traq, i_trar, i_trat, i_trav, i_traw, i_tray, i_trba, i_trbb, mobf, mobp1, t_virb5, trau, virb4* | *dfrA12, aadA2, cmlA1, ANT(3'')-IIa, qacH, sul3, mefB, tetA* | *acrF, acrE, acrS, bacA, tolC, CRP, mdfA, kdpE, mdtN, mdtO, mdtP, H-NS, mdtH, mdtG, mdtM, marA, cpxA, emrR, emrA, emrB, baeR, baeS, mdtC, mdtB, mdtA, ugd, mdtE, gadW, gadX, ampC (class C β-lactamase), acrD, mphB, eptA, dfrA12, evgA, evgS, emrK, emrY, pmrF, yojI, fosA3, acrA, acrB, ampH, msbA* |
| F542C | Chicken | *f_trae, f_traf, f_trag, f_trah, f_trak, f_tral, f_tran, f_trau, f_trav, f_traw, mobf, mobh, virb4* | *marA, tetA* | *emrR, emrA, emrB, CRP, baeR, baeS, mdtC, mdtB, mdtA, cpxA, emrY, emrK, evgA, evgS, Escherichia_coli_mdfA, H-NS, yojI, pmrF, msbA, mdtG, mdtH, mdtP, mdtO, mdtN, mdtE, mdtF, gadW, gadX, acrS, acrE, acrF, kdpE, acrA, acrB, ampH, ugd, mdtM, mphB, acrD, ampC (class C β-lactamase), tolC, eptA, bacA* |
| F379B | Chicken | *f_trab, f_trae, f_traf, f_trag, f_trah, f_trak, f_trau, f_trav, f_traw, mobh, mobp1, t4cp1, t_virb11, t_virb2, t_virb6, t_virb8, t_virb9, virb4* | *qnrS1, mdtM, mdtP, mdtO, ANT(3'')-Iia, qacH, sul3, tetA* | *msbA, mdfA, fosA3, mdtG, mdtH, fosA4, dfrA14, eptA, ugd, mdtA, mdtB, mdtC, baeS, baeR, emrB, emrA, emrR, H-NS, ampC (class C β-lactamase), acrD, mphB, acrS, acrE, acrF, tolC, gadX, gadW, mdtF, mdtE, emrY, emrK, evgA, evgS, bacA, yojI, pmrF, kdpE, CRP, mdtM, acrA, acrB, ampH, mdtP, mdtO, marA, ANT(3'')-IIa, qacH, cpxA* |
| F543B | Chicken | *f_trae, f_traf, f_trag, f_trah, f_trak, f_tral, f_tran, f_trau, f_trav, f_traw, i_trae, i_trai, i_trak, i_tral, i_tran, i_trao, i_trap, i_traq, i_trar, i_trat, i_trav, i_traw, i_tray, i_trba, i_trbb, mobf, mobp1, t_virb1, t_virb3, t_virb5, t_virb6, t_virb8, t_virb9, trau, virb4* | *mphA, sul1, tetA, sul2, aadA5, dfrA17, mdtM, mdfA, mdtH, mdtG* | *mphB, acrD, ampC (class C β-lactamase), emrA, emrR, acrS, acrE, APH(3'')-Ib, APH(6)-Id, H-NS, baeR, baeS, mdtC, mdtB, mdtA, pmrF, yojI, tolC, mdtN, mdtO, mdtP, evgS, evgA, emrK, emrY, CRP, mdtM, bacA, marA, Escherichia_coli_mdfA, kdpE, acrA, acrB, msbA, cpxA, gadX, gadW, mdtF, mdtE* |
| F480A | Chicken | *f_trae, f_trag, f_trah, f_trak, f_tran, f_trav, i_trae, i_trai, i_trak, i_tral, i_tran, i_trao, i_trap, i_traq, i_trar, i_trat, i_trav, i_traw, i_tray, i_trba, i_trbb, mobf, mobp1, t_virb11, t_virb2, t_virb6, t_virb8, t_virb9, trau, virb4* | *aadA2, cmlA1, ANT(3'')-IIa, qacH, APH(3")-Ib, APH(6)-Id, fosA3, blaCTX-M-14, sul3* | *ampH, acrB, acrA, kdpE, acrD, mphB, bacA, tolC, acrF, acrE, acrS, cpxA, ugd, mdtA, mdtB, mdtC, baeS, baeR, yojI, pmrF, evgS, evgA, emrK, emrY, eptA, mdfA, mdtN, mdtO, mdtP, marA, emrR, emrA, emrB, mdtH, mdtG, msbA, CRP, mdtE, mdtF, gadW, gadX, blaTEM-1, mdtM, ampC (class C β-lactamase), dfrA1, sul2, H-NS, emrE* |
| F166A | Beef | *f_trae, f_traf, f_trag, f_trah, f_trak, f_tral, f_tran, f_trau, f_trav, f_traw, mobf, mobq, virb4* | *kdpE, sul2, APH(3'')-Ib, APH(6)-Id, dfrA5, tetA, blaTEM-1* | *evgS, evgA, emrK, emrY, pmrF, yojI, baeR, baeS, mdtC, mdtB, mdtA, acrS, acrE, acrF, mdtM, mdtG, cpxA, marA, msbA, H-NS, CRP, mdtE, mdtF, gadW, gadX, mdfA, mdtP, mdtO, mdtN, dptA, acrA, acrB, ampH, emrB, emrA, emrR, mphB, acrD, ampC (class C β-lactamase), tolC, bacA* |
